# The declining affordability of dental care in New Zealand from 1978 to 2023

**DOI:** 10.1111/cdoe.12998

**Published:** 2024-08-16

**Authors:** Ryan Gage, Jonathan Broadbent, William Leung, Martin Lee, Trudy Sullivan

**Affiliations:** ^1^ Department of Public Health University of Otago Wellington New Zealand; ^2^ Sir John Walsh Research Institute University of Otago Dunedin New Zealand; ^3^ Community Dental Service Te Whatu Ora—Waitaha Canterbury Christchurch New Zealand; ^4^ Department of Preventive and Social Medicine University of Otago Dunedin New Zealand

**Keywords:** dental services research, economics, oral health policy, time series

## Abstract

**Background:**

In the early 2020s, nearly half of New Zealand adults reported that cost of treatment had prevented them from accessing dental care, with higher rates among Māori, Pasifika and individuals living in the most deprived areas. Unaffordable dental care may be explained by a rise in dental service fees over time relative to personal income, as documented in New Zealand between 1978 and 1993. However, there have been no contemporary estimates in New Zealand of how the affordability of dental care has changed. The aims of this study were to analyse the change in dental treatment fees and the personal income of New Zealanders from 1978 to 2023 and to explore differences in affordability of dental care by ethnicity.

**Methods:**

Average fees for dental treatments were sourced from surveys completed by practising New Zealand dentists. Earnings (from 1978) and personal income data (full population from 2000 and by ethnicity from 2008) were sourced from Statistics NZ and NZ Official Yearbooks. Inflation‐adjusted changes in average fees, weekly personal earnings and income were calculated as a percentage change from 1978 levels for fees and earnings and from 2000 for personal income.

**Results:**

For the five dental treatments with data available from 1978 to 2023, fees increased in the range of 75%–236%, while earnings increased by 46% over the same period. Fees for other treatments (with data available from 1981 to 2009) similarly increased and mostly surpassed changes in earnings. From 2008 to 2023 the overall increase in personal income (about 21% across all ethnic groups) kept pace with the rising cost of most treatments. However, due to persistent income inequalities, in 2023, Māori and Pasifika would need to spend a higher proportion of their weekly income (approximately 16% and 23% respectively) to receive the same dental treatments as NZ Europeans.

**Conclusions:**

Fees for dental treatments have risen markedly in recent decades, more sharply than the price of other goods and services.

## 1 INTRODUCTION

While universal free dental care is available in Aotearoa/New Zealand (NZ) for children up to the age of 17 years, adults can only access a small range of publicly funded dental services and those vary widely across the country.^1^ Nearly half (44%) of adult participants in the 2018/2019 NZ Health Survey reported that cost of treatment had prevented them from accessing care for a dental problem during the past year.^2^ In contrast, only 13% had not received needed general practitioner (medical) services in the past year due to cost, and only 5% had not filled a prescription due to the cost.^2^ The odds of having unmet need for dental care are 1.3 times greater for Māori (95% CI 1.2, 1.3), 1.2 times greater for Pasifika (95% CI 1.1, 1.3) and 1.5 times greater for the most deprived New Zealanders relative to the least deprived (95% CI 1.3, 1.7).^2^

After exiting the publicly funded dental system at age 18 years, levels of untreated dental caries rise markedly and peak in the mid‐20s,^3^ before slowly dropping back as increasing tooth loss leaves fewer teeth available for caries attack.^4^ For those aged 18 years or over, public funding is very limited. NZ health policymakers are aware there is a problem with access to dental care for low‐income adults,^5^ and some, but not all, NZ regional public dental services fund or provide emergency dental treatment for low‐income adults.^6^ For beneficiaries, the Ministry of Social Development also provides discretionary special needs grants and benefit advances for dental treatment.^7^

Private expenditure on dental services in NZ was estimated at NZ$912 million in 2008,^8^ increasing to NZ$1.6 billion in 2017 (NZ$912 million in 2008 = NZ$1.05 billion in 2017).^9^ If these figures are accurate, they represent a 52% increase in private system expenditure in a single decade. It is difficult to ascertain the veracity of these estimates however, due to the lack of available evidence, indicating how little is known in this area. Comparatively, the spend on publicly funded oral health services was estimated at NZ$185 million in 2008^8^ and NZ$199 million in 2014/2015^10^ (NZ$185 million in 2008 = NZ$209 million in 2015), showing in real terms a decrease in publicly funded oral health expenditure.

One way to estimate changes in affordability of dental care is to investigate the change over time in dental service fees and personal income. In NZ, this has only been reported once previously, by Devlin and Stanley in 1994. They explored the trends in dental treatment costs and household incomes between 1978 and 1993.^11^ In a period when average household incomes remained relatively static, the cost of dental care increased considerably, resulting in less affordable dental care. A limitation of this earlier work was the absence of ethnicity subgroup analysis. The study was also conducted at a time when the regulation of dental practice was less stringent and protocols such as the autoclave sterilization of reusable instruments were not consistently done.^12^ There have been considerable changes to dentistry since then, including the establishment of the Health Practitioners' Competence Assurance Act, recertification requirements for oral health practitioners and new and advanced dental techniques.^13^

An ideal health care service should be equitable, accessible and meet whānau (family) needs. NZ's oral health care services could do better on all three domains. There is a clear need to adapt the NZ oral health care system to better meet the needs of the population. To enable sound policy development for public funding of dental care, a better understanding of the affordability of dental care is needed.

This study aims to advance the line of research instigated by Devlin and Stanley^11^ by providing contemporary estimates of how the affordability of dental care has changed over time. Specifically, the aim of this study is to analyse the changes in dental treatment fees and personal income between 1978 and 2023 and examine differences by ethnicity.

## 2 METHODS

### 2.1 Dental treatment fees

Dental treatment fees were sourced from the NZ Dental Association (NZDA) survey, which collects data on the fees charged by practising dentists for core dental services. The survey was first conducted by NZ's Ministry of Health in 1975 to collate information on dentist incomes and the cost of providing dental care.^14^ The survey subsequently evolved into the NZDA fee survey in 1978 and since then has been conducted at 1‐ or 2‐year intervals. From the 1970s to 1990s, participation in the survey ranged from 30% to 50% of practising dentists^11^ with the sample considered sufficiently representative of national data. In past NZDA fee surveys, up to 500 responses were received for the fees questions, representing approximately one in five practising dentists (average participation rate 19%).^15^ Specific details on the representativeness of participating dentists are not available, as only the information on average and quartiles of dentist fees (aggregated nationally and by geographic region) is made publicly available by NZDA. The ‘average’ data were used for the analyses reported in this paper. Treatment fees were measured in terms of the average fee charged per item, including Goods and Services Tax (GST). The GST rate increased twice over the period of analysis, from 10% to 12.5% in 1989 and from 12.5% to 15% in 2010.

### 2.2 Average earnings and income

Data on earnings and income were sourced from Statistics NZ.^16^ Earnings data before 1989 were obtained from NZ Official Yearbooks.^17^ To calculate changes in earnings from 1978 to 2023, the average gross weekly earnings for those aged 15+ years in paid employment were used. These data were not disaggregated by ethnicity. Personal income among ethnic subgroups, the preferred measure, only became publicly available in 2008.^16^ This measures the average gross weekly income from all sources and includes individuals who were not in paid employment. It is lower, on average, than the average gross weekly earnings metric used to analyse trends from 1978.

### 2.3 Consumer price index

Mean dental treatment fees and average earnings and income for each year were adjusted for inflation using the consumer price index (CPI) for all CPI groups. These groups include: food, housing, health, recreation/culture, education, communication, clothing, transport, alcohol/tobacco, household contents/services and miscellaneous.^16^

### 2.4 Analyses

Cross‐sectional tables plotting changes in dental service fees and income over time (in 2023 NZ$) were compiled in Microsoft Excel 365. Changes in dental fees for individual treatments were estimated as a percentage change from 1978 to 2023 and from 2008 to 2023 (when ethnicity‐specific income data became available). The summed cost and percentage change for four core treatment scenarios were calculated for the same time periods. These treatment scenarios were chosen as being illustrative of different types of treatment.

1. Routine check‐up and scale: routine examination and simple prophylaxis scaling (15 min). Average cost in 2023: NZ$255.
2. Extraction: simple extraction (×2), acrylic partial denture. Average cost in 2023: NZ$1699.
3. Restorative and basic rehabilitative care: routine examination and simple prophylaxis, scaling (15 min), 2 surface composite, multi‐surface composite, single root canal, all ceramic crown. Average cost in 2023: NZ$3525.
4. Basic restorative and extraction care: routine examination and simple prophylaxis, scaling (15 min), multi‐surface composite, a simple tooth extraction and an additional simple extraction, metal partial denture. Average cost in 2023: NZ$3355.

To investigate the relative change in dental services over time, the annual CPI values for dental services (a subgroup of health services) were compared against the CPI for health services and health subgroups (pharmaceutical products, medical services and health insurance). These data were available from 1999 for health services and from 1981 for dental services and health insurance. As CPI data are published quarterly, an average CPI value was calculated for each year.

## 3 RESULTS

**FIGURE 1:**
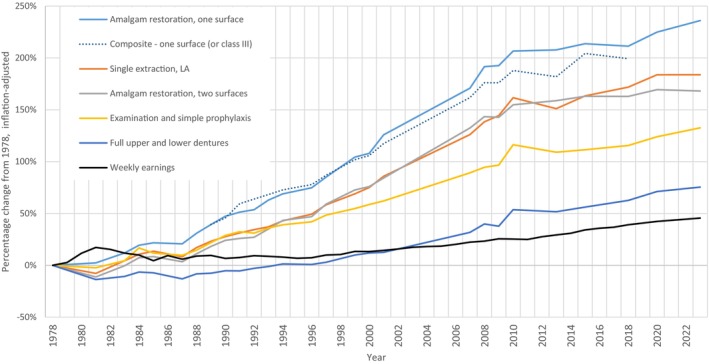
Percentage change in inflation‐adjusted dental fees and weekly earnings^a^ from 1978 levels.

Five dental treatments had data available from 1978 (examination and simple prophylaxis, single extraction, amalgam restoration (one surface), amalgam restoration (two surfaces) and full upper and lower dentures). The inflation‐adjusted percentage change in average fees for these procedures from 1978 to 2023 is shown in Figure 1. Also shown in Figure 1 is the estimated inflation‐adjusted average weekly earnings for all New Zealanders and, for comparison, the percentage change in average fees for one‐surface composite restorations (added to the NZDA fee survey in 1989). New Zealanders' inflation‐adjusted weekly earnings remained fairly static from 1978 to the late 1990s, and then increased gradually, reaching 46% more than 1978 levels by 2020. In contrast, inflation‐adjusted fees for the index dental procedures increased in the range of 75%–236%. The greatest increases were for one‐surface amalgam fillings (236% increase) and single tooth extractions (184%). The smallest increase (75%) in fees was for complete upper and lower dentures.

Annual changes in the average fees for other treatments (added to the NZDA Fee survey from 1981 to 2009) are presented in Table 1. Also reported in Table 1 are the annual changes in weekly earnings from 1978, the annual changes in weekly income (from all sources) from 2008 and the percentage changes in treatment fees and income from 2008 (when ethnicity‐specific income data became available) to 2023. When comparing 2023 fees to the first year treatment service fees were available (between 1981 and 2009, depending on treatment), the majority of service fees increased at a rate higher than inflation. Three exceptions were all ceramic crowns (3% decrease from 2007 to 2023), implants for single teeth (20% decrease from 2001 to 2023) and the surgical component of implants (20% decrease from 2007 to 2023).

**TABLE 1:** Weekly income and dental treatment fees (including GST) from 1978 to 2023 (2023).

	1978	1981	1983	1985	1987	1989	1990	1993	1996	1997	2000	2001	2007	2008	2009	2010	2013	2015	2018	2020	2023	% change 2008 to 2023	% change 1978 to 2023
Statistics NZ income/CPI data																							
CPI, all groups	160	245	306	375	491	552	586	615	664	672	698	716	834	867	886	906	963	978	1019	1053	1240		
Average weekly earnings^a^	1062	1245	1187	1110	1124	1163	1133	1154	1140	1167	1203	1214	1299	1310	1334	1330	1373	1424	1477	1511	1546	18	46
Average weekly income (Total population)											775	800	910	898	895	890	895	959	1049	1022	1090	21	
Average weekly income (NZE)														954	952	948	956	1022	1121	1089	1133	19	
Average weekly income (Māori)														786	753	738	747	791	875	898	980	25	
Average weekly income (Pacific)														689	666	650	617	709	800	780	898	30	
Dental fee survey data																							
Examination and simple prophylaxis	68	67	71	76	75	83	88	93	97	101	108	111	129	133	134	148	143	145	147	153	159	20	133
Scaling per 1/4 h						74	76	89	91	96	100	102	116	127	120	126	115	113	110	101	96	−25	
Complex periodontal per 1/2 h						155	178	202	194	207	229	218	244	256	245	264	234	233	219	211	217	−15	
Panoramic X‐ray						79	78	87	86	90	94	97	107	112	111	114	111	113	111	113	105	−6	
Single extraction, LA	103	95	107	116	111	126	131	141	153	162	180	191	232	244	251	268	257	270	279	291	291	19	184
Each additional tooth extracted, LA						43	47	58	65	77	91	95	144	147	153	157	151	156	168	183	193	31	
Root filling—single root									450	485	528	565	721	773	787	838	816	874	895	945	960	24	
Root filling—molar (3 roots)											949	1008	1213	1287	1290	1351	1310	1359	1406	1477	1481	15	
Amalgam restoration, one surface	60	61	67	73	72	83	88	98	105	111	124	135	162	174	175	183	184	188	186	194	201	15	236
Amalgam restoration, two surfaces	94	83	94	102	97	111	117	127	138	149	165	173	218	229	228	240	243	247	247	253	252	10	168
Composite—one surface (or Class III)						88	93	113	121	129	146	156	195	207	207	218	212	232	228			10^b^	
Glass ionomer—one surface (or class III)						81	85	101	108	116	130	137	163	176	178	183	179	185	203			16^b^	
Composite—(class II)											204	218	268	279	277	287	282	290	299	309	308	10	
Composite—multisurfaced						132	144	173	190	207	274	291	352	363	364	376	363	379	392	393	378	4	
Composite crown						209	218	268	291	312	371	410	464	476	482	501	487	488	497	509	502	5	
Fissure sealant—one tooth						47	49	52	52	54	59	61	83	90	83	86	86	91	94	92	99	10	
All Ceramic crown													1675	1739	1733	1781	1722	1719	1704	1715	1624	−7	
Porcelain laminate veneer							736	895	943	980	1097	1129	1384	1497	1480	1553	1500	1513	1541	1557	1504	0	
PFM crown											1404	1420	1587	1650	1652	1697	1671	1672	1702	1698		3^c^	
Full upper and lower dentures	1914	1652	1708	1776	1664	1768	1814	1891	1930	1970	2142	2153	2522	2677	2636	2941	2904	2988	3113	3277	3358	25	75
Acrylic partial—one tooth						492	508	611	629	666	688	710	834	886	896	948	927	986	1008	1072	1117	26	
Metal partial—one tooth						1210	1219	1461	1520	1588	1703	1727	1986	1993	2022	2089	2125	2150	2204	2260	2238	12	
Denture reline—heat processed						323	330	381	386	393	427	428	465	480	491	498	514	527	521	522	517	8	
3‐unit anterior bridge						2993	3047	3518	3569	3684	3886	3954	4502	4611	4592	4761	4552	4540	4615	4515	4157	−10	
Implant, single tooth—superstructure											3758	3549	3670	3938	3682	3712	3609	3458	3439	3366	3001	−24	
Implant (surgical component)													3884	3750	3716	3728	3599	3598	4108	3324	3091	−18	
Hygienist—1/2 h rate											107	114	125	134	127	133	126	129	134			0^b^	
Target hourly rate		328	361	386	364	412	426	444	444	469	498	513	563	592	575	623	591	588	571	569	541	−9	
Treatment scenarios																							
Routine check‐up and scale													245	260	255	274	257	257	257	254	255	−2	
Extraction													1298	1375	1397	1485	1442	1527	1566	1654	1699	24	
Restorative and basic rehabilitative care													3261	3414	3416	3556	3441	3520	3548	3616	3525	3	
Basic restorative and extraction care													2959	3008	3044	3164	3154	3213	3299	3382	3355	12	

*Note*: Restorative and basic rehabilitative scenario: Routine examination and simple prophylaxis, scaling (15 min), 2 s composite, multi‐surface composite, single root canal, all ceramic crown. Extraction scenario: single extraction (×2), acrylic partial denture. Basic restorative and extraction care scenario: Routine examination and simple prophylaxis, scaling (15 min), multi‐surface composite, single extraction, metal partial denture. Routine check‐up and scale scenario: Routine examination and simple prophylaxis, scaling (15 min).

^a^ For those in paid employment.

^b^ Calculated to 2018.

^c^ Calculated to 2020.

When focusing on the period 2008–2023, the fees for most dental treatments (19 of the 27) increased more rapidly than inflation. The greatest increases were in additional tooth extractions (31% increase), acrylic partials (26% increase) and complete upper and lower dentures (25% increase). A small number of services decreased in cost over the time period: complex periodontal work (15% decrease), implants for single teeth (24% decrease), implants (surgical component) (18% decrease), 3‐unit anterior bridges (10% decrease), all ceramic crowns (7% decrease), panoramic x‐rays (6% decrease) and scaling (25% decrease). Dentists' target hourly rate decreased by 9% from 2008 to 2023.

Average inflation‐adjusted weekly income (from all sources) for the total population of NZ increased by 21% from 2008 to 2023. This was similar to the increase in weekly earnings over this period (18%). The 18%–21% change in weekly income (from all sources) and weekly earnings exceeded the inflation‐adjusted change in 21 of the 27 treatments. Increases in inflation‐adjusted weekly income from 2008 to 2023 were higher for Māori (25% increase) and Pasifika (30% increase) than NZ European (19% increase). However, despite these increases, considerable inequalities in average weekly income by ethnicity persisted in 2020 (NZ$1133 for NZ European, NZ$980 for Māori and NZ$898 for Pasifika).

For the four treatment scenarios, inflation‐adjusted treatment costs increased from 2008 to 2023 (Table 1). An exception was the routine‐check up and scale scenario, for which average treatment costs decreased 2%; this was less than the increase in inflation‐adjusted income for this period (for all subgroups). Treatment costs for the extraction scenario increased 24%. For the restorative care scenario, average treatment costs increased 3%; again, this was less than the increase in inflation‐adjusted income for the total population (21%). For the low‐income restorative care scenario, average treatment costs increased 12%, which was less than the increase in inflation‐adjusted income (19%).

**FIGURE 2:**
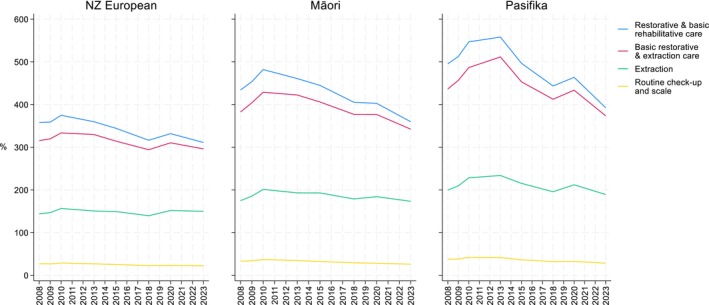
Treatment scenarios as a % average weekly income by ethnicity from 2008 to 2023 (2023 NZ$).

Figure 2 plots the cost of each treatment scenario as a percentage of average weekly income, by ethnic group, over the 2008–2023 period. When compared to NZ Europeans, the percentages are approximately 16% higher for Māori and 23% higher for Pasifika, indicating that these groups would have to pay a higher proportion of their income to receive the same dental treatment. For example, in 2023, the routine check‐up and scale scenario would cost NZ Europeans 22.5% of their weekly income, compared to 26.0% for Māori and 28.4% for Pasifika.

**FIGURE 3:**
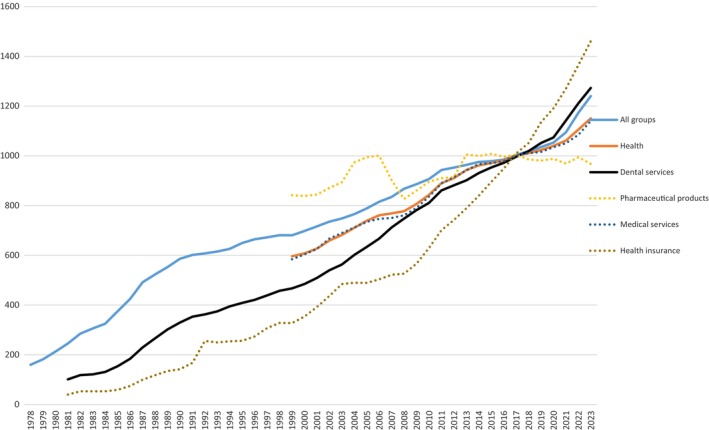
Annual changes in the CPI for dental services compared with the CPI for all groups and health services.

Similar to Devlin and Stanley's results,^11^ the CPI for dental services increased at a faster rate than the CPI for all groups and the CPI for most health services (Figure 3). For example, from 2008 to 2023, the CPI for dental services increased at nearly twice the rate (70% increase) of the CPI for all groups (43%), and more than the CPI for health services (48%).

## 4 DISCUSSION

Dental service fees in NZ have continued to increase at a higher rate than inflation, outstripping price increases in other goods and services, as illustrated in the CPI statistics for dental vs other health services. Although increases in personal income have kept up with increases in treatment fees from 2008 to 2023, the results show a considerable decline in dental care affordability since 1978, with only a small number of treatments decreasing in cost or remaining static in this period. A number of changes in the practice of dentistry may help to explain these shifts, and below we discuss some of the likely reasons for this, along with some of the limitations and implications of this research.

Large increases in fees occurred for glass ionomer restorations, root fillings and dentures. Glass ionomer cement restorations are used differently to the past; these are now available in capsulated form and are often a definitive or ‘permanent’ restorative material rather than an interim one, owing to improvements in the material properties of glass ionomer products.^18^ Increases in the cost for dentures may be due to rising quality standards and the shrinkage of the discipline; fewer complete dentures are now provided due to marked reductions in the rate of edentulism^19^ with fewer dentists offering removable prosthodontic care than in the past. Standards of care in endodontic treatment have also become more rigorous and complex,^20^ likely resulting in greater time and material costs for providing endodontic care.

Caries rates, tooth loss and periodontal disease have declined in NZ since the 1970s, while the dental workforce has grown. In 1973 there were 34 dentists per 100 000 population;^11^ this had risen to 62 per 100 000 (aged 15+ years) by 2019.^21^ Our finding of increasing fees supports that of a recent report that described how Norwegian dentists who feel they have too few patients are able to counteract any adverse effect on their incomes by providing more dental services and raising their fees.^22^

Increases in the fees for periodontal services and scaling occurred at a rate lower than most other services, and this may be due to changes in the profession. First, a shift towards greater emphasis on a preventive model of care means that periodontal care has become more mainstream, and a routine procedure carried out for many patients. Second, competition may be acting to help suppress fees for this service. The introduction and growth of the oral health therapy/dental hygiene workforce means that there are now many more health practitioners available to do such work. Increases in the fees for implant superstructures (abutments and crowns) were also lower than other services, and this may be due to implant components being more readily available using ‘stock’ components, rather than being custom designed and manufactured. There has also been considerable growth in the number of suppliers of these products, with numerous companies now supplying implant components in NZ, up from a very limited number of companies 15 years ago. (Furthermore, it is now possible to order such components from offshore companies.) These are issues that warrant further investigation.

There are a number of possible explanations for dental service fees increasing at twice the rate of other goods and services. Changes in regulation have led to stricter cross‐infection control and instrument re‐processing requirements, which takes additional time and necessitates the purchase and maintenance of specialized equipment and associated increases in the cost of dentistry. There may have been shifts in expectations with respect to the retention of teeth, and patients may expect more of dental care.^23^ Furthermore, it is possible that the changing ownership model of dental practices, with an ever‐rising number of dental practices being owned by corporations rather than dentists, may be leading to rising costs—there are now more people with which to share the profits (e.g. practice owners and shareholders) rather than just the clinician and their team who provide the care. Recent qualitative research has reported how corporate interests may influence the provision of dental care,^24^, ^25^ and future health services research is needed to investigate the extent to which this is an issue in NZ and internationally.

Māori and Pasifika need to spend a higher proportion of their weekly income to receive the same dental treatment as NZ Europeans. These data align with higher rates of unmet need for dental care among Māori and Pasifika.^2^ Reducing inequalities in access to affordable dental care could be achieved by expanding the capacity of hospital dental services and standardizing available services, together with strategies to reduce population oral health burden.^1^ Policy should also focus on the upstream drivers of inequalities in disposable income, such as higher unemployment and lower home ownership (more income being spent on rent) among Māori and Pasifika,^26^ which further worsens affordability of dental care. These findings are also consistent with European research that has reported how changes in dental fees can impact access to dental care^27^; that research recommended that fee regulation could help to address this issue, and the findings from this study lend further support to this assertion.

Limitations of this study include the singular focus on dental service fees as a measure of how dental practices have changed over time. While data on dentist incomes and costs are collected through the NZDA Cost of Practice survey, owing to privacy concerns and commercially sensitive information, these data were unavailable. Furthermore, due to differences in how NZ population income data are collected over time, the only data available throughout the entire period of interest were average weekly earnings among those in paid employment. This measure overestimates the disposable income available to spend on goods and services such as dental care. While a measure of personal income was also used across all sources by ethnicity, these data were only available from 2008.

## 5 CONCLUSION

Fees for dental treatments have risen markedly in recent decades, more sharply than the price of other goods and services. While an equivalent increase in personal incomes occurred in the 2008–2023 period, affordability has declined markedly since 1978, with the relative rise in fees for most dental services nearly doubling that of weekly earnings. This decline is particularly concerning for Māori and Pasifika, given inequalities in income by ethnicity, which have persisted since ethnicity‐specific income data became available in 2008. An ideal health care service should be equitable, accessible and meet family/whanau needs. To achieve these ends, NZ oral health policy should focus on delivery of population‐level preventive interventions alongside enhancing access to basic and emergency dental care services for disadvantaged population groups.

## CONFLICT OF INTEREST STATEMENT

The authors certify that the research is original, not under consideration elsewhere, and free of conflict of interest.

## Data Availability

Data on dental fees were obtained from the New Zealand Dental Association. These fees data have been published previously by the New Zealand Dental Association, but only data from the most recent survey are currently available on the Association's website. Data from previous surveys are replicated in Table 1 of this manuscript. https://www.nzda.org.nz/public/resources/nzda‐fee‐survey. Data on earnings and income were sourced from Statistics NZ for the years 1989–2023. https://infoshare.stats.govt.nz/infoshare/. Earnings data before 1989 were obtained from New Zealand Official Yearbooks. https://www.stats.govt.nz/indicators‐and‐snapshots/digitised‐collections/yearbook‐collection‐18932012/.
